# Physical inactivity, non-participation in sports and socioeconomic status: a large population-based study among Norwegian adolescents

**DOI:** 10.1186/s12889-020-09141-2

**Published:** 2020-06-26

**Authors:** Ove Heradstveit, Siren Haugland, Mari Hysing, Kjell Morten Stormark, Børge Sivertsen, Tormod Bøe

**Affiliations:** 1 Regional Centre for Child and Youth Mental Health and Child Welfare, NORCE Norwegian Research Centre, Nygårdsgaten 112, 5008 Bergen, Norway; 2 Center for Alcohol & Drug Research, Stavanger University Hospital, Stavanger, Norway; 3 Department of Psychosocial Sciences, Faculty of Psychology, University of Bergen, Bergen, Norway; 4 Department of Health Promotion and Development, University of Bergen, Bergen, Norway; 5 Department of Health Promotion, Norwegian Institute of Public Health, Bergen, Norway; 6 Department of Research & Innovation, Helse Fonna HF, Haugesund, Norway; 7 Department of Mental Health, Norwegian University of Science and Technology, Trondheim, Norway

## Abstract

**Background:**

Physical activity in adolescence is found to promote both immediate and long-term health, as well as school- and work performance. Previous studies suggest that parental socioeconomic status (SES) may influence the level of activity, although the results are inconsistent. The objective of this study was to examine the overall level of low physical activity/sports participation and the associations with parental SES and adolescent school program in a population-based study of older adolescents.

**Methods:**

The youth@hordalandy study, a large population-based study in Hordaland county, Norway, conducted in 2012, included 10,257 adolescents aged 16–19 years (53% girls). Physical activity was examined by self-reported overall activity, and participation in organized team- and individual sports. Predictor variables were parental SES measured by youth self-reports of family economic well-being, parental education and work affiliation and self-reported current high school program (vocational versus general studies). Age, ethnicity, and family structure were included as covariates.

**Results:**

Girls who reported lower parental education had small, but significant higher risk for physical inactivity and non-participation in individual sports compared with their counterparts who reported higher family education (RRs ranging from 1.04 to 1.12, *p* < 0.01). There were some, but inconsistent, evidence of an increased risk for physical inactivity and non-participation in sports among those (and particularly boys) with lower family economic well-being. Parental work status was largely unrelated with physical inactivity/non-participation in sports. Adolescents in vocational studies had a small but significantly increased risk of physical inactivity and non-participation in sports compared with individuals in general studies (RRs ranging from 1.03 to 1.05, all *p* < 0.05).

**Conclusions:**

We found some evidence of a social gradient for lower physical inactivity and non-participation in sports for adolescents. Although effect sizes were small, vocational studies was the most robust correlate for physical inactivity/non-participation in sports among the SES-variables, while the corresponding associations with parental education and family economic well-being differed by gender.

## Background

Physical activity in adolescence is associated with several immediate and long-term positive effects on health [1]. Physical activity may facilitate learning and school performance [2], as well as general well-being [3]. Despite such well-known benefits, level of physical inactivity is generally high in Western countries [4]. As with health outcomes in general, physical activity seems to be socially distributed, and individuals from the lower end of the social hierarchy are less likely to be physically active compared to their more advantaged peers (e.g. [5–8]). However, the literature on how socioeconomic status (SES) relates to physical activity during adolescence is far from uniform [9], and there is a pronounced need to improve our understanding of the correlates of physical inactivity in this age group [10].

In adult populations, physical inactivity is associated with low level of education, but largely unrelated to income (for a review, see [11]). During adolescence physical activity may still be influenced by parental factors. From this perspective, it is of interest to examine the level of physical activity of adolescents in relation to parental SES. In a cross-national review of adolescents aged 13 to 18 years, the majority of studies showed that higher parental SES was positively associated with higher levels of physical activity [9], whereas 42% percent of the included studies reported no or an opposite relationship. The authors highlighted several potential explanations for these inconsistencies. For example, different measures of SES (such as income and parental education) may have different saliency in regards to adolescent physical activity levels across countries.

Importantly, varying measures of exercise may also have contributed to the previous inconsistent findings on how SES correlate with adolescent physical activity levels [9]. In Norway, adolescent sports are largely organized through memberships in teams or sport clubs. Participation may require active parental participation in coaching and voluntary work, and increasing economic and instrumental support in the teen-age years. Unlike many other countries, there are no national programs for extracurricular school-based sports. These factors may produce differences according to socioeconomic background, as well as diversity based on cultural values and ethnicity. In the present study we therefore included both an overall, generalized measure of frequency of weekly physical activity, as well as two measures of participation in organized sports. The use of multiple measures, enables a more nuanced examination of how SES is associated with physical activity [9, 12, 13].

A range of variables may potentially affect physical activity levels during adolescence, and it is therefore important to account for counfounding variables in the study of associations between SES and physical activity [10]. Previous research has demonstrated that self-rated health vary considerably across ethnic groups within a country [14], and that ethnic background correlates with physical inactivity [15, 16]. Age is also of importance, as there is a considerable drop in membership rates in organized sports during mid- and late adolescence, in particular for girls and ethnic minority groups [17]. Additionally, a general decline of physical activity levels during adolescence is a consistent finding in the literature [18]. A range of parental influences, such as family cohesion, parent-child communication and parental engagement, affect adolescent physical activity levels [19, 20], while also being associated with family structure (e.g. single-parent versus two-parent households) [21]. It is also found that family structure correlate with higher body mass index and potentially lower physical activity among youth [22]. Therefore, it will be useful to account for ethnicity, age and family structure in analyses of associations between SES and physical activity.

Increased knowledge of the correlates of physical activity according to SES may identify factors that can be modified to promote equity in health outcomes. Based on a large population-based study among Norwegian adolescents aged 16–19 years, the aims of the present study were to investigate physical inactivity and non-participation in organized sports, in relation to parental SES and high school program, while also taking into account sex, age, ethnicity, and family structure.

## Methods

### Study design and setting

The current cross-sectional and population-based study used data from the youth@hordaland-study of adolescents aged 16 to 19 years in the county of Hordaland in Western Norway, which targeted adolescent health, lifestyle and service use. One year prior to the survey, all included questionnaires were piloted and refined in a single school. Adolescents in upper secondary education (both general education and vocational studies) received information by email followed by an SMS reminder, and they were given time during regular school hours to complete the questionnaire. For those not at school during the allocated school completion, the questionnaire could be completed at other times at their convenience during the study period, and some schools also arranged catch up days. Students not enrolled in school received information by postal mail to their home address and could complete the questionnaire online. We also contacted hospitals and institutions to accommodate for completing the questionnaire. The web-based questionnaire was administered using computers, and a teacher was present to organize the data collection and to ensure confidentiality. Adolescents and school personnel could direct queries to survey staff that was available on phones during the study period. Adolescents not in school received information by postal mail and could complete the questionnaire online.

### Participants

All adolescent born from 1993 to 1995 were invited to participate in the survey during spring 2012. An invitation to participate was sent to a total of 19,430 adolescents, of which 10,257 (53%) took part in the study.

### Instruments

#### Demographic information

Sex and year of birth were based on the personal identity number in the Norwegian National Population Registry. The adolescents were asked who they were currently living with, and a range of response categories were available, including biological mother, biological father, stepmother, and stepfather. We used this variable to determine whether they were living in a single- or two-parent household. Ethnicity was based on adolescent self-reported country of origin, and categorized as either Norwegian, from a country in the European union or in the European Economic Area (EU/EEA), of from a country not in the EU/EEA (non-EU/EEA).

#### Measures of SES

##### Perceived economic well-being

Perceived economic well-being was assessed by the following question: “Compared to others, how would you rate your family’s economic situation”. The response options were “Poorer than others”, “Equal to others”, or “Better than others”. Similar questions have previously been used with adolescents as a measure of SES [23, 24].

##### Parental education levels and work affiliation

The adolescents were asked to indicate the level of parental education using the options “elementary school”, “high school, vocational”, “high school, general”, “college/university less than four years”, “college/university four years or more” and “don’t know”. This variable was re-categorized into basic (i.e. elementary school), intermediate (i.e. high-school levels), higher (i.e. college/university levels). Based on this, a variable denominating the highest education in the household was created (e.g., if the mother had “higher” education and the father had “intermediate” education, the educational level of the household was “higher”). Cases reporting “don’t know” to both items of parental level of education were omitted from the final analyses.

Adolescents were asked by an open-ended question to indicate parental work affiliation and work category. This was coded according to ISCO-08 classification [25]. “Being in work” was defined as parents with a work ISCO code or parents reported to be students. The “out-of-work” category consisted of parents who were not working outside the home (including sick leave, disability pensions, as well as unemployed parents and homemakers). Based on this information, we created a variable that differentiated between individuals in whom (i) both parents worked, (ii) both parents were out-of-work, or (ii) one parent worked and the other was out-of-work.

##### Adolescent school program

The Norwegian school system consists of elementary school (ages 6–13), lower secondary school (ages 13–16), and upper secondary school / high school (ages 16–19). The latter is divided into general and vocational studies. Whereas general studies prepare students for pursuing higher education, such as studies at university or college, vocational studies focus on practical skills and a specific trade. High school program may be seen as a proxy for future SES, as few students in vocational training programs pursue higher academic education. Despite the particular importance of physical strength and tolerance for repetitive muscle strain needed for many vocational careers, previous studies suggest that students in vocational programs have overall high levels of health risk factors, including physical inactivity [26, 27]. In the present study, all adolescents indicated their current school program which was categorized as either “general studies” or “vocational studies” based on the Norwegian high-school system.

#### Physical activity and sports participation

In accordance with previous publications [28, 29], frequency of physical activity was measured by the following question: “Over the past seven days, how many days were you physically active at a minimum of 60 minutes daily?” The response categories were number of days (0 through 7) [30]. A dichotomous variable for “physical inactivity” was created (1 = “0-1 times a week”, 0 = “2-3 times a week” to “6-7 times a week”). Sports participation was measured by questions about frequency of organized individual- (e.g. swimming, gymnastics, martial art) or team sports (e.g. soccer, handball, or hockey) using the response options “Don’t engage in this activity”, “2-3 times a month or less”, “About once a week”, or “Twice a week or more often”. Non-participation variables were created for both individual and team sports (1 = “Don’t engage in this activity” = 1, 0 = “2–3 times a month or less”, “About once a week”, and “Twice a week or more often”).

### Statistical analyses

All analyses were conducted using R version 3.3.3 for Mac (R Core Team, 2016). Descriptive analyses of the sample was conducted using t-tests for independent samples and Pearson chi-square tests to indicate sex differences across the included variables (Table 1). Associations with physical inactivity and non-participation in individual- and team sports were investigated using regression analyses with the package *rms* (Harrel, 2017). Due to significant interaction effects with sex for several relevant variables, final models were conducted separately for boys and girls. Three separate binomial regression models were run (Table 2) with dichotomized variables predicting physical inactivity and non-participation in individual- and team sports, respectively. In the regression models, all predictors were entered simultaneously to investigate their relative influence on physical inactivity or non-participation in sports. In addition, age, ethnicity and family structure were included as covariates in the models. The categorical predictor variables were dummy coded in the logistic regression models, thereby allowing comparisons of a specific level of the categorical variables to a reference level from the same variable. As outcomes were common, a binomial regression using the “log”-link to obtain relative-risk ratios was attempted, but did not converge. Instead, a modified Poisson regression model was fitted to the data (Zou, 2004). The modification involves robust error variance adjustment by use of a sandwich estimator in R to correct for overestimation of error when applied to binomial data (Zeileis, 2004; 2006).

**Table 1:** Descriptive characteristics of the sample

	Girls	Boys	*p*
	(*N* = 4317)	(*N* = 3766)	
Age	M = 17.49 (SD = 0.85)	M = 17.45 (SD = 0.84)	0.042
Ethnicity			0.099
Non-EU/EEA	117 (2.7%)	107 (2.9%)	
EU/EEA	117 (2.7%)	75 (2.0%)	
Norway	4022 (94.5%)	3540 (95.1%)	
Family economic well-being			< 0.001
Poorer than others	339 (8.0%)	218 (5.9%)	
Equal to others	2909 (68.6%)	2310 (62.4%)	
Better than others	994 (23.4%)	1171 (31.7%)	
Family structure			0.778
Not single parent	3241 (83.9%)	2787 (83.7%)	
Single parent	620 (16.1%)	544 (16.3%)	
Family education			0.412
Basic	238 (5.5%)	187 (5.0%)	
Intermediate	1633 (37.8%)	1463 (38.8%)	
Higher	2446 (56.7%)	2116 (56.2%)	
Family work status			0.893
Out-of-work (both parents)	55 (1.3%)	43 (1.2%)	
Out-of-work (one parent)	243 (5.7%)	211 (5.8%)	
Work (both parents)	3950 (93.0%)	3394 (93.0%)	
School program			< 0.001
General studies	2745 (63.9%)	2028 (54.1%)	
Vocational studies	1552 (36.1%)	1719 (45.9%)	
Physical activity			< 0.001
0–1 times a week	1156 (27.9%)	720 (20.6%)	
2–3 times a week	1550 (37.4%)	1090 (31.1%)	
4–5 times a week	964 (23.3%)	925 (26.4%)	
6–7 times a week	474 (11.4%)	767 (21.9%)	
Individual sports participation			< 0.001
Don’t engage in this activity	2495 (60.1%)	2146 (61.1%)	
2–3 times a month or less	374 (9.0%)	324 (9.2%)	
One day a week	502 (12.1%)	275 (7.8%)	
Twice a week or more	777 (18.7%)	769 (21.9%)	
Team sports participation			< 0.001
Don’t engage in this activity	3013 (72.7%)	2056 (58.3%)	
2–3 times a month or less	244 (5.9%)	264 (7.5%)	
One day a week	239 (5.8%)	285 (8.1%)	
Twice a week or more	647 (15.6%)	924 (26.2%)	

*EU* European Union. *EEA* European Economic Area

**Table 2:** Associations between SES and physical inactivity and non-participation in sports^1^

	Physical inactivity				Non-participation in individual sports				Non-participation in team sports			
	Girls		Boys		Girls		Boys		Girls		Boys	
	*RR* (95% CI)	*p*-value	*RR* (95% CI)	*p*-value	*RR* (95% CI)	*p*-value	*RR* (95% CI)	*p*-value	*RR* (95% CI)	*p*-value	*RR* (95% CI)	*p*-value
**Parental SES**												
Family economic well-being (Better than others is reference)												
Equal to others	1.02 (0.99–1.05)	0.090	**1.03 (1.00–1.05)**	**0.030**	**1.03 (1.01–1.06)**	**0.005**	**1.03 (1.01–1.06)**	**0.002**	1.01 (0.99–1.03)	0.361	**1.02 (1.00–1.05)**	**0.043**
Poorer than others	1.04 (0.99–1.09)	0.069	1.04 (0.99–1.10)	0.102	1.03 (0.99–1.07)	0.194	**1.05 (1.00–1.09)**	**0.032**	**1.05 (1.02–1.08)**	**0.003**	**1.08 (1.03–1.12)**	**< 0.001**
Family education (Higher is reference)												
Intermediate	**1.05 (1.03–1.07)**	**< 0.001**	1.00 (0.98–1.02)	0.944	**1.04 (1.02–1.06)**	**< 0.001**	1.00 (0.98–1.02)	0.919	0.98 (0.97–1.00)	0.053	1.00 (0.98–1.02)	0.781
Basic	**1.12 (1.06–1.17)**	**< 0.001**	1.01 (0.96–1.06)	0.736	**1.08 (1.04–1.12)**	**< 0.001**	1.04 (0.99–1.09)	0.051	1.02 (0.99–1.05)	0.260	1.00 (0.95–1.05)	0.990
Parent work status (Both parents working is reference)												
Out-of-work (one parent)	1.01 (0.97–1.06)	0.590	1.03 (0.98–1.08)	0.252	1.03 (0.99–1.07)	0.113	1.02 (0.98–1.06)	0.397	1.00 (0.97–1.03)	0.995	**1.06 (1.02–1.10)**	**0.003**
Out-of-work (both parents)	1.09 (0.99–1.19)	0.078	1.10 (0.99–1.22)	0.065	1.03 (0.96–1.11)	0.386	0.99 (0.90–1.08)	0.786	0.96 (0.89–1.03)	0.223	0.99 (0.90–1.09)	0.866
**Adolescent SES**												
School program (General studies is reference)												
Vocational studies	**1.03 (1.01–1.05)**	**0.016**	**1.05 (1.03–1.08)**	**< 0.001**	**1.04 (1.02–1.06)**	**< 0.001**	**1.04 (1.01–1.06)**	**0.001**	**1.04 (1.02–1.05)**	**< 0.001**	**1.05 (1.03–1.07)**	**< 0.001**

*SES* Socioeconomic status. *RR* Relative risk. *CI* Confidence interval

^1^All analyses are additionally adjusted for age, ethnicity, and family structure

The proportion of missing data ranged from 0.5% for school program, to 11% for family structure, with a mean of missing at 3% across the included variables. Missing data was handled with multiple imputation with the R package *mice* [31]. Ten imputed datasets were created, and the results from the regression analyses were pooled across the imputed datasets. In single-parent families, and in other cased where information about one parent was missing, the parental education level and work affiliation of the non-missing parent was used. The reporting of the present study followed the STROBE guidelines [32].

## Results

### Descriptives

The mean age of the participants was 17 years, and the sample included more girls (52.7%, *n* = 5401) than boys (47.3%, *n* = 4856). There were significant sex differences in perceived family economic well-being (*p* < 0.001). Specifically, somewhat more girls than boys reported their economic well-being to be poorer than others (8% for girls versus 6% for boys) and more boys reported to be better off than others (23% for girls versus 32% for boys). Around 16% of the sample lived in single-parent households, and most of the adolescents reported that their parents had intermediate or higher education, and that they were working. More boys than girls reported enrollment in vocational studies (*p* < 0.001). Physical inactivity was more often reported by girls than boys (*p* < 0.001), and girls were also less likely to be engaged in individual and team sports compared to boys (*p* < 0.001). See Table 1 for descriptive characteristics of the sample.

### Physical inactivity

In the analysis predicting physical inactivity, there were significant associations with school program for both girls and boys. Adolescents in vocational studies were more likely to be physically inactive (RRs = 1.03–1.05, all ps < 0.05) compared to general studies. There were also some sex specific main effects of family economic well-being for boys, and of family education and ethnicity for girls. Boys who rated their family economic well-being as ‘equal to others’ were somewhat less likely to be physically inactive (RR = 1.03, *p* < 0.05) relative to peers who perceived their economic well-being as better. Girls whose parents had intermediate or basic education were less physically active (RRs = 1.05 to 1.12, all ps < 0.001) relative to their peers with more educated parents.

### Non-participation in individual sports

There were significant associations with family economic well-being and school program for both sexes for non-participation in individual sports. Adolescents who described their family economic well-being as either equal to others (RR = 1.03, *p* < 0.01) or poorer than others (boys only; RR = 1.05, *p* < 0.05) were less likely to participate in individual sports, compared with adolescents who perceived their economic well-being as better than others. Adolescents in vocational studies (RRs = 1.04, all ps ≤ 0.001) were less likely to participate in individual sports compared with adolescents in general studies. Girls whose parents had intermediate or basic education were also more likely to be non-participants in individual sports (RRs = 1.04 to 1.08, all ps < 0.001) relative to their peers with higher educated parents.

### Non-participation in team sports

Adolescents who reported to be poorer than others were more likely to be non-participants in organized team sports (RRs = 1.05 to 1.08, all p s < 0.01), as were boys who reported family economic well-being equal to others (RR = 1.02, *p* < 0.05) relative to peers with better family economic well-being. Adolescents in vocational studies (RRs = 1.04 to 1.05, all ps < 0.001) were also less likely to be engaged in team sports.

## Discussion

Current high school program, a proxy for future SES, was the most consistent correlate of physical inactivity and non-participation in organized sports across the sexes. Specifically, adolescents in vocational studies had a small but significantly increased risk for physical inactivity and non-participation in sports compared with students in general education. In addition, lower parental education reported by girls, and lower family economic well-being reported by boys, correlated with physical inactivity and non-participation in sports.

### Adolescent school program

The finding of a higher risk for physical inactivity and non-participation in sports among vocational school students resonate with previous contributions. Haug and colleagues [26] noted that a significant proportion of vocational school students engaged in multiple health risk behaviors, such as tobacco smoking, hazardous drinking, and physical inactivity. However, comparisons with non-vocational students were not made in their study. Similar to the present findings, a Swedish study of 16 year-olds found a lower level of physical activity in students in vocational programs compared to theoretical programs [33]. The association was partly explained by parental SES. A study by Vereecken and colleagues [27] reported a higher risk for a range of unhealthy behaviors within vocational students; however, their study did not include physical activity, and did not adjust their analyses for SES. Our findings add new insights to the saliency of adolescents with low education as a particular risk factor for physical inactivity and non-participation in sports, these associations were present in models that adjusted for parental SES and relevant co-variates. Although we cannot make firm conclusions on the mechanisms involved, the reduced level of physical activity and sports participation in vocational school students may be partly explained by a school-day with longer hours at work sites compared with adolescents attending general studies. Future studies are needed to expand our understanding of the reduced physical activity levels in this group.

### Parental SES

In the present study, we found that poorer *economic well-being* predominantly had independent associations with physical inactivity and non-participation in sports for boys, while a similar although less pronounced pattern was identified for girls. These findings lend some support to studies that highlight family income as a particularly consistent correlate of adolescent physical activity [34, 35]. However, our models accounted for other SES-variables and relevant co-variates as well, and were therefore informative of the unique contribution of family economy on physical inactivity/non-participation in sports. Our findings thus demonstrate independent associations between family economic well-being and physical inactivity/non-participation in sports for boys, while these associations were less robust among girls.

On the other hand, our findings suggest that *parental education* was a somewhat more robust correlate of physical inactivity and non-participation in individual sports for adolescent girls compared with boys. These findings lend some support to previous studies which reported an association between high parental education and both organized sports participation [36, 37] and physical activity [37, 38]. Of particular note, a Finnish study of adolescents found low parental eduation to be an independent risk factor for physical inactivity and non-participation in sports also after adjusting for economic disadvantage, in line with our findings [37]. However, previous findings have pointed to significant correlations between parental education and physical activity across sexes [37], these findings were not replicated in our study. A previous German study among 11–17 year olds showed that parental education was more strongly associated with physical activity than were occupation and income [39]. Our findings support this notion to some extent for girls.

Despite the abovementioned gender differences in associations between economic well-being and parental education (i.e., different aspects of parental SES) and physical inactivity/non-participation in sports, it should be noted that effect sizes were overall small, and we advise to interpret these findings with caution. Our data do not allow for any clear interpretation of the mechanisms involved in these heterogeneous associations, and future studies are encouraged to further explore how measures of parental SES relates to physical activity/sports participation and potentially differ be gender.

A third indicator of parental SES, *parental work status*, was largely unrelated to physical inactivity and non-participation in sports in the present study. These findings add to previous studies that largely have shown parental occupational status to be unrelated to adolescent physical activity (for a review, see 34).

### Strengths and limitations

The present study has several strengths. We used a large population-based sample, from on a region of Norway considered to be nationally representative in many respects. It is considered a strength to include measures of both overall physical activity and sports participation, while we also applied multiple measures of SES along with relevant co-variates. To our knowledge no previous studies have compared adolescent physical inactivity and non-participation in sports across vocational and general studies, and our study thus presents novel results. The study also has some limitations. Our measure of physical inactivity did not fully account for category, frequency, duration and intensity [9], and were not specifically operationalized to separate adolescents that meet WHO recommendations for physical activity from those who do not. Also, membership in a gym is not specified as an organized sport. Validated and more detailed questionnaires assessing physical activity and sports participation would have been preferable and improved the manuscript in this respect. However, it should also be noted that the focus of the present study was on non-participation in sports and physical inactivity, which is likely to have been properly measured by the response categories of the applied questions. Also, the inclusion of three different measures may be interpreted as a strength, as these measures tap into several important aspect of physical activity in this age group. Similarly, the present study measured SES by several self-reported items, and not with objective measures of income or registry-based parental education records, which would have strengthened this study. Subjective measures of SES, such as perceived economic well-being, are commonly used indicators of socioeconomic status of adolescents [23], but may be prone to bias [40]. On the other hand, the measure of perceived economic well-being suffers less from non-response compared to traditional indicators such as income and wealth and corresponds fairly well with objective indicators, such as family income [24]. The response rate was around 53%, raising the possibility of a non-participation bias. National data show that in 2012, 92% of all adolescents in Norway aged 16 to 18 years attended upper secondary school, compared to 98% in the current study. While we do not have available data on the non-responders in this survey, previous rounds of the Bergen Child study (the same population as the current study) have reported higher rates of mental health problems among non-attenders [41]. Previous research has suggested that associations between variables are fairly robust to non-participation bias [41], but the strength of these associations could be either under- or overestimated, something our data do not allow to fully disentangle.

## Conclusion

The present study found some evidence of a social gradient for lower physical inactivity and non-participation in sports for adolescents. Although effect sizes were small, vocational studies was the most robust correlate for physical inactivity/non-participation in sports among the SES-variables, while the corresponding associations with parental SES differed by gender. Future studies are needed to enhance our understanding of how associations between parental SES and physical activity/sports participation potentially differ by gender.

This study based on a large sample in late adolescence shows that vocational education appeared to be the most consistent correlate of physical inactivity and non-participation in sports. Several indicators of parental SES, including family economic well-being and parental education, were to some extent associated with physical inactivity and non-participation in sports. Particularly, low parental education reported by girls, and low family economic well-being reported by boys, correlated with physical inactivity and non-participation in sports.

## Data Availability

The Norwegian Health research legislation and the Norwegian Ethics committees require explicit consent from the participants in order to transfer health research data outside of Norway. For the Bergen Child study, which constitutes the data for the current analyses, ethics approval was also contingent on storing the research data on secure storage facilities located in our research institution, which prevents us from providing the data as supplementary information or to transfer it to data repositories. Individual requests for data access should be sent to bib@norceresearch.no.

## Abbreviations

SES: Socioeconomic status
EU / EEA: European union / european economic area
ISCO: International standard classification of occupations
STROBE: Strengthening the reporting of observational studies in epidemiology
